# Complement’s involvement in allergic Th2 immunity: a cross-barrier perspective

**DOI:** 10.1172/JCI188352

**Published:** 2025-05-01

**Authors:** Sarah A. Thomas, Stephane Lajoie

**Affiliations:** 1 W. Harry Feinstone Department of Molecular Microbiology and Immunology, Johns Hopkins Bloomberg School of Public Health, Baltimore, Maryland, USA.; 2 Department of Otolaryngology-Head and Neck Surgery, Johns Hopkins School of Medicine, Baltimore, Maryland, USA.

## Abstract

Type 2 (Th2) allergic diseases are chronic conditions characterized by a Th2-polarized immune response to allergens. These diseases can be categorized by affected barrier sites: skin (atopic dermatitis, allergic contact dermatitis), gut (food allergy), and respiratory tract (e.g., asthma, chronic rhinosinusitis). The global prevalence of Th2 allergic diseases has increased the need for a deeper understanding of their pathophysiology. Several associations have been identified between genetic variants in the genes encoding components of the complement system and allergic disease. Moreover, levels of several complement proteins are elevated in patients with allergy. Experimental evidence demonstrates that the complement system plays a critical role in the development of these diseases across barrier sites. While site-specific differences exist in the complement components involved, key pathways, particularly C3 and C5, are prominent across the skin, gut, and lung.

## Complement: a brief overview

The complement system is an intricately interconnected cascade of over 50 soluble and cell-bound proteins that function as a part of the innate immune system (1). Originally thought of as solely liver derived, local sources of complement within tissues are now appreciated as critical regional coordinators of immunity (2, 3). Complement activation occurs through three primary pathways: the classical, lectin, and alternative pathways. The classical pathway is initiated by the binding of C1q to immune complexes or pathogen-associated molecular patterns, while the lectin pathway is triggered by the recognition of specific carbohydrate residues on microbial surfaces. The alternative pathway can be activated through pathogen recognition or tissue damage and through spontaneous C3 hydrolysis, which occurs at low levels even in the absence of infection (1). Despite these distinct activation mechanisms, all three pathways converge at the formation of C3 and C5 convertases, multisubunit protein complexes that cleave C3 and C5 into bioactive fragments with various functions. Complement was once thought to function exclusively in the extracellular space, but more recent evidence has revealed additional intracellular effects (4–6). Intracellularly, it is thought that complement proteins such as C3 and C5 are cleaved by proteases such as cathepsins (4, 7), resulting in intracellular bioactive fragments of complement that regulate cellular processes such as metabolism, autophagy, and the elimination of intracellular pathogens (8–10). While first described over a century ago in the context of microbiological insults, it has become clear that complement is an integral part of diverse biological processes, including Th2 immunity — the focus of the present Review.

## Complement involvement in allergy

The prevalence of allergic diseases is increasing globally. In the United States alone, 24 million people have asthma (11), and over 100 million people experience symptoms of allergy (12, 13). With consideration of their chronicity and their associated socioeconomic burden, atopic diseases thus constitute a substantial public health challenge (14, 15). In susceptible individuals, allergic diseases arise due to a pathophysiological Th2-polarized immune response mounted against a harmless insult, the allergen (16). This aberrant immune response is precipitated by the interaction between the environment (allergen) and the body at barrier surfaces, i.e., the skin, gut, and lungs. Allergic diseases are numerous and mechanistically diverse, though they share a common etiological theme in a Th2-polarized immune response. There are abundant data illustrating the phenomenon known as “atopic march,” whereby an initial sensitization in infancy lays the foundation for allergic disease later in childhood in a skin-to-gut-to-lung axis: atopic dermatitis (AD) manifests first, followed by food allergy (FA), and then finally asthma (17). The close relationship between the allergic diseases is evident by their high comorbidity; for example, patients with AD have been reported to have 3–4 times the odds of having a second atopic disease (18). Thus, while there is utility in framing discussions in the context of the affected barrier, it should be advised that allergic diseases are not discrete entities but rather manifestations of often multitissue pathology in susceptible individuals.

Thus, the immunologic mechanisms of allergic diseases are vastly complex and beyond the scope of this discussion. Briefly, the canonical view of allergic sensitization, or the process of generating allergen-specific IgE antibodies, begins with allergen uptake by DCs at barrier surfaces (19). DCs then migrate to regional lymph nodes to present allergen to naive CD4^+^ T cells, triggering polarization into Th2 cells. Discussions around the mechanisms driving allergic disease often revolve around adaptive immunity. However, it is important to emphasize that innate immune mechanisms such as complement are critical in shaping the tone and magnitude of type 2 responses.

**Figure 1:**
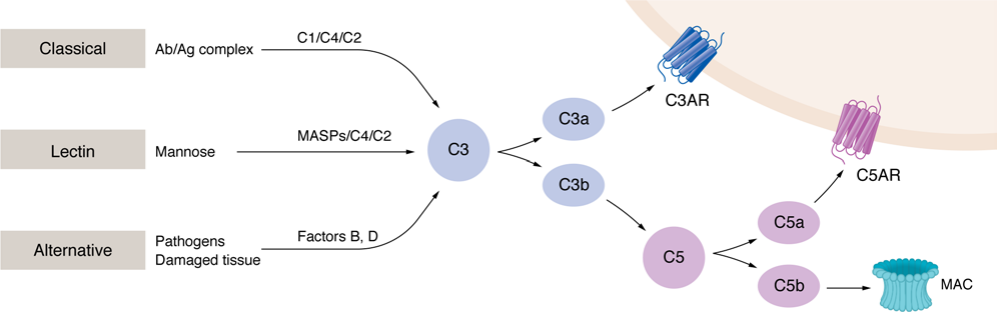
Pathways of the complement system. A basic overview highlighting the complement components relevant to the discussion in this Review of complement in allergic disease. Ag, antigen; MASP, mannan-binding lectin serine protease; MAC, membrane attack complex.

The complement system has emerged as an integral component of myriad immunological processes, including in type 2 (Th2) immunity (20, 21). The complement system has numerous ties to initiating and propagating allergic inflammation (Table 1). A basic overview of the complement components relevant to the subsequent discussion is provided in Figure 1. Perhaps the most well-cited components are the anaphylatoxins, complement fragments C3a, C4a, and C5a (22). These complement fragments are soluble inflammatory proteins named for their ability to elicit anaphylaxis, a systemic, life-threatening allergic reaction (22, 23). However, as will be discussed, far more complement components than the anaphylatoxins are implicated in allergy. The subsequent sections will outline connections between complement and allergic diseases according to the barrier site affected.

## Genetic clues connecting complement to allergic diseases

Allergic diseases are multifactorial and are thought to result from a combination of environmental and genetic drivers. Numerous genetic variations in complement components have epidemiological ties to allergic diseases, though it is worth noting that most data focus on allergic asthma; other Th2 allergic diseases are not as well represented. SNPs in complement genes have been associated with the likelihood of having allergy. For example, SNPs in C3, C3AR1 (encoding a C3a receptor), and C5 have been positively associated with bronchial asthma in Japanese children and adults (24–26), Caribbean adults (27), and French Canadian women (28). In a case-control study of Italian children, the frequency of C5 polymorphism was increased among individuals with asthma (29). Interestingly, protective C5 polymorphisms have been identified in patients with AD, with patients exhibiting the C5 rs366510 SNP having reduced allergen-specific serum IgE against common allergens such as Dermatophagoides pteronyssinus (house dust mite [HDM]) (30). Other studies have identified polymorphisms in complement-encoding genes that correlate with poor outcomes, such as an association between C3 rs448260 and more frequent asthma hospitalizations (31). Together, these data provide an evidentiary foundation for the role of complement in the pathophysiology underpinning allergic diseases. Indeed, a growing body of evidence is illuminating the close relationship between complement and allergic skin, gut, and lung diseases.

## Complement and skin allergy

AD is a type 2 allergic disease of the skin, and it presents clinically as dry, itchy, and red skin (32). AD commonly manifests in infancy and affects approximately 20% of children and 10% of adults globally (33). Fundamentally, AD represents a failure of barrier integrity at the skin, whereby barrier permeability potentiates allergen interaction with local immune cells, allowing for allergic sensitization (32). AD represents the first step in the atopic march and is thought to be a precursor to gut and airway allergies via epicutaneous sensitization (34, 35).

Keratinocytes are the most abundant cell type in the epidermis and function to promote barrier integrity as well as act as immune sentinels in the skin (36). While there is evidence that human keratinocytes express an array of complement components — including C3, C3aR, and complement inhibitors such as complement factor I (CFI) and complement factor H (CFH) — the majority of this evidence is derived from nonallergic contexts, indicating a potential homeostatic role for complement signaling in the skin (37–39). However, aberrant complement activation is thought to play a role in AD. A polymorphism in C3AR1 is associated with children with asthma who have manifestations of AD (26). C3 has been shown to play a role in instigating allergic inflammation in the skin. Some of the first work to identify a link between AD and complement showed that C3 and its cleavage product, C3a, were elevated in skin biopsies and blood of patients with AD (40–42). Interestingly, these changes were not isolated only to eczematous regions and were instead seen throughout the skin (43), thus providing early evidence supporting the notion of allergic diseases as systemic rather than purely localized disorders. In one study, epicutaneous OVA challenge induced skin and blood eosinophilia in wild-type but not C3-deficient mice (43). Moreover, C3-deficient mice had reduced Th2 cytokine expression at the site of exposure, indicating a dampening of the Th2 response. In the same study, in vitro OVA challenge of splenocytes from OVA-sensitized mice elicited increased production of IL-4, IL-5, IL-13, and IFN-γ, an effect not observed in C3 deficiency.

Dysregulated complement also drives other manifestations of type 2–driven skin inflammation. Aberrant anaphylatoxin-driven mast cell and basophil degranulation is thought to worsen disease in patients with chronic urticaria (CU) (44). In line with this, studies of CU show elevated levels of serum C3 and C4 (45). Furthermore, elevated C3 is thought to be a factor in patients with CU who display resistance to anti-IgE treatment (46). Similarly, overzealous C3a and C5a signaling on skin mast cells is thought to enhance experimental cutaneous allergy, an IgE-dependent mouse model of type I hypersensitivity (47). Other studies examining contact allergic dermatitis in mice showed either a dispensable (48) or a protective (49) role for C3 in response to irritant-induced dermatitis (using toluene-2,4-diisocyanate). In contrast, C5a signaling through C5aR1 promotes inflammation in models of allergic dermatitis (50). Conversely, C5a signaling through C5aR2 (also known as C5L2) protects against oxazolone-induced allergic contact dermatitis, as it is thought to prevent aberrant activation of the C5aR1 (50), possibly via inhibition of C5aR1/β-arrestin–mediated initiation of the ERK1/2 signaling cascade (51). Metal exposures can also drive skin allergies; nickel and cobalt allergies are the most common and often manifest as contact dermatitis (52–54) and even airway allergy (55, 56). They have been shown to activate C3 and complement factor B (CFB) in human plasma, while relatively nonallergenic metals like barium, copper, and zinc did not (57). Thus, dysregulated complement in the skin may act as a sensitizer to the downstream development of Th2 responses.

## Complement and gut allergy

FA affects approximately 10% of US adults (58), among whom nearly half report allergies to multiple foods (59). Anaphylaxis, a severe and potentially fatal manifestation of FA, is also thought to be driven by aberrant complement activation. Levels of C3a are elevated in individuals with anaphylaxis and correlate with the severity of anaphylaxis (60). Peanut allergy, one of the most common and severe types of FA, is mainly mediated by complement. Peanut extract has been shown to activate complement and induce rapid C3a accumulation in vitro and in vivo (61, 62). In animals, administration of peanut extract i.v. causes rapid shock and death in a C3-dependent, IgE-independent manner (62). Mechanistically, this occurs through C3-induced release of histamine and platelet-activating factor, which activates macrophages, basophils, and mast cells. It is thought that the combination of C3 mobilization and IgE activation drives the full magnitude of anaphylactic symptoms. Conversely, foods that do not mobilize C3, like milk and egg, show little ability to induce shock (62). Notably, nonfoods known to cause severe anaphylaxis in humans, such as Hymenoptera (bee, wasp, etc.) venom and penicillin derivatives, also cause complement activation (63–65). Similar to C3, C5a/C5aR1 signaling is pathogenic in an OVA model of FA (66). C5a, especially in male mice, is necessary to drive the entirety of the FA phenotype, including shock-associated hypothermia (66). Altogether, complement may act as a powerful adjuvant in certain manifestations of severe allergies.

## Complement and airway allergy

Dysregulated complement levels are observed in Th2-mediated allergic diseases of the respiratory system, impacting both the lower (e.g., asthma) and upper (e.g., chronic rhinosinusitis, allergic rhinitis) respiratory tracts (67). At the systemic level, serum C3, C3a, and C4a are elevated (67–70) and positively correlated with asthma severity outcomes among adults (31, 67) and children (71). In a large study of 101, 029 individuals, elevated circulating levels of C3 were correlated with elevated IgE and blood eosinophils levels and were associated with asthma exacerbation and hospitalization (31). Anaphylatoxin accumulation is also observed in the allergic airways. C5a is elevated in asthmatic sputum compared with that in control sputum (72), and C3a and C5a were found to be increased in the bronchoalveolar lavage (BAL) fluid after segmental allergen challenge in patients with mild asthma. Moreover, C3 protein measured in exhaled breath was associated with uncontrolled asthma (73). In addition, both anaphylatoxin receptors (C3aR and C5aR) were shown to have elevated expression in cases of allergic rhinitis (74), nasal polyps (75), and fatal asthma (76), laying the groundwork for a local role for complement in the pathogenesis of allergic airway diseases (77, 78). Allergic diseases largely implicate dysregulated levels of C3, C5, and their corresponding anaphylatoxins. However, other, less studied components of complement, like CFH (79), and terminal components of complement, like C6, C7, and C8, have been found in some studies to be elevated in patients with asthma (73, 80, 81). Furthermore, CFB, a driver of the alternative pathway of complement, has been shown to drive allergic manifestations in a mouse model of asthma (82).

## C3 and C5 in airway allergy

The association between complement and allergy was first appreciated in the early 1950s with the observation that anaphylatoxins were potent inducers of histamine release (83), a major driver of immediate symptoms of allergy. In fact, intradermal injection of C3a in healthy volunteers drives wheal and flare reactions in minutes (84), which were partly inhibited by antihistamine administration. This discovery is particularly relevant to asthma, as C3a is known to exacerbate bronchoconstriction, which is thought to involve the activation of mast cells (85, 86). However, the importance of C3a-induced mast cell degranulation in the pathogenesis of asthma is debated (87–91). Mast cells originating from the yolk sac (MC_TC_) are rich in granules containing tryptase, chymase, and carboxypeptidase. They reside in connective tissues, fat, and the skin’s submucosa, near blood vessels, lymphatics, and neurons. C3aR is highly expressed by MC_TC_. Human skin mast cells display high responsiveness to anaphylatoxins, including C3a, leading to histamine release (92, 93). In contrast, bone marrow–derived mast cells (MC_T_), mainly located in respiratory and gastrointestinal mucosal tissues, primarily contain tryptase and express little C3aR. Reports show that human lung mast cells do not respond to C3a or C5a (90, 91, 94). While mast cells in human lungs are overwhelmingly (90%) of the MC_T_ variety (95), it is conceivable that the relatively small proportion of MC_TC_ cells in the human lung may respond to C3a in disease, such as those that infiltrate airway smooth muscle bundles (96). However, the presence of C3a-responsive lung mast cells in humans remains to be formally demonstrated. Finally, antihistamines, while important for the treatment of allergic rhinitis, cannot treat asthma and are only considered optional adjunctive therapy to alleviate secondary symptoms for disease management (97). This suggests that C3a promotes asthma largely through mast cell–independent mechanisms. Thus, while the importance of C3a-induced mast cell degranulation in the pathogenesis of asthma may be controversial, it is clear that C3a signaling is crucial for airway allergy. Various asthma triggers — such as HDM, ozone, cigarette smoke, viruses, and pollutants — activate C3, drive type 2 inflammation, and promote airway hyperresponsiveness (AHR) in a C3-dependent manner in mouse models (78, 98–106).

The primary sources of C3 during allergy and the mechanisms by which it drives Th2 immunity remain areas of active exploration. C3, once thought to be solely derived from the liver and distributed systemically via circulation, is now known also to be produced locally at mucosal surfaces, including the airways (2, 3). C3 is found in various pulmonary cell types at steady state, including the epithelium, and in a variety of immune cells in both mice and humans (98, 107, 108). C3 mRNA and protein are upregulated in primary human airway epithelial cells in response to allergen, leading to the accumulation of C3a (98). Mechanistically, it is now understood that C3a can signal to a range of immune cells beyond mast cells. Notably, C3a has been shown to enhance DC activities, such as antigen uptake and expression of costimulatory molecules in vitro (109). However, C3aR signaling in DCs does not appear to play a large role in HDM-induced allergic responses in vivo (110). Recent findings reveal that C3a signaling contributes to type 2 immune responses by promoting expansion of group 2 innate lymphoid cells (ILC2s) in an HDM-induced mouse model of airway allergy (98). C3a, which is notably elevated in individuals with uncontrolled asthma (67, 68), has also recently been implicated in promoting the formation of neutrophil extracellular traps (NETs) (111, 112), a process believed to contribute to the pathogenesis of more severe forms of the disease (113–115). C3a may also contribute to disease via direct signaling to epithelial cells. C3a was shown to induce the expression of MUC5AC, a key component of mucin in cultured mouse bronchial epithelial cells (116), and repress vitamin D metabolism in human upper airway epithelial cells (117), which is part of a well-known antiallergic pathway.

The role of C5 in allergy is more complex and nuanced than that of C3. C5 and its anaphylatoxin, C5a, had traditionally been perceived as purely proinflammatory. However, a seminal study utilizing several genetic crosses of mouse strains with either resistance or susceptibility to AHR discovered C5 as a locus of protection (118). Some mouse strains (A/J, AKR/J) are naturally C5-deficient due to gaining a stop codon in the C5 gene. These were more susceptible to OVA-induced AHR than C5-sufficient strains (C57Bl6/J, BALB/cJ, C3H/HeJ) (118). Further work has shown that C5a signaling could have a dual role in allergy. During allergen sensitization, C5a protects against the development of Th2 inflammation and AHR but enhances disease in animals with established allergy (119, 120). The protective effect of C5a is thought in part to be driven by the coinhibitory molecules PD-L1 and PD-L2 on plasmacytoid DCs (pDCs) (121), previously shown to promote tolerance in models of airway allergy (122). In contrast, C5a signaling to adoptively transferred allergen-pulsed bone marrow–derived DCs drives Th2 inflammation in the airways (123). Interestingly, deletion of C5aR in the myeloid compartment in LysM^Cre^ mice had no significant effect on OVA-induced lung allergy (124). This suggests that nonmyeloid C5aR^+^ cells confer the protective or deleterious effect of C5a during allergy. Because pDCs do not express LysM, this implies that the protective role of C5a during the sensitization phase of allergy is partly mediated by pDCs. This may translate in humans, as pDCs also respond to C5a (125). Conversely, the cell type that drives the proallergic effects of C5a on established disease remains more nebulous. While LysM^Cre^ marks almost all monocytes, macrophages, DCs, and neutrophils, only one-fifth of eosinophils are affected by LysM^Cre^-mediated targeting (126). Recent work suggests that airway allergen exposure elicits a population of induced eosinophils, contrasting with resident eosinophils, expressing elevated levels of intracellular C5aR. In this context, C5a does not function as an eosinophil recruitment factor; it promotes the degranulation of these activated eosinophils. This was shown to drive AHR but not other manifestations of allergy, like mucus secretion or Th2 cytokine production (127). Thus, the question of which other C5a-responsive cell(s) exacerbate type 2 inflammation remains to be understood.

Another mechanism through which aberrant complement activation may promote allergy is by inhibiting Tregs. C3a and C5a have been identified as negative regulators of mouse and human Treg function (128, 129). This effect may be through direct signaling to Tregs as in vitro stimulation of CD4^+^ cells. The absence of both C3aR and C5aR signaling synergistically leads to TGFβ1-dependent Treg autoinduction (128). This translated to humans, as both C3aR and C5aR antagonism also induced Treg differentiation of CD4^+^ T cells. In an Aspergillus model of allergic airway inflammation, allergen challenge of C3ar1-deficient mice resulted in increased frequency of CD4^+^Foxp3^+^ cells in the secondary lymphoid organs and lungs but not thymus (130). Wild-type mice receiving a bone marrow transplant from C3ar1-deficient mice also showed an increased frequency of CD4^+^Foxp3^+^ cells in the spleen, suggesting C3aR-mediated Treg suppression was primarily attributed to hematopoietic cells. Similarly, exposure to chitin, an integral component of arthropods (mites, cockroaches, etc.), in animals sensitized to fungal allergy drove C3-dependent Th2 cells and a concomitant abrogation of Tregs (99).

In addition to regulating Th2 responses, both C3a and C5a have been shown to act as critical regulators of Th17 cells in the context of allergy. In models of more severe disease, Th17 cells are coelicited alongside Th2 cells, where C3a has been shown to promote Th17 cells. This is thought to happen via C3a enhancing the production of IL-23, as measured in allergen-exposed lung homogenates and mouse bone marrow–derived DCs (101, 131). This is supported by findings demonstrating C3a as a driver of IL-23 in human blood-derived monocytes, leading to increased Th17 responses (132). However, others have found that C3a could inhibit Th17 airway responses in animals sensitized to an Aspergillus protease/OVA mix (130). In contrast to C3a, C5a impairs the development of Th17 cells by inhibiting IL-23 and promoting IL-10 from mouse splenic and bone marrow–derived DCs (101, 133). Thus, in addition to regulating type 2 responses, it is thought that C3 and C5a may alter disease severity by modulating allergen-induced Th17 responses.

It is intriguing to draw parallels between the role of complement in pulmonary fibrosis and allergy, as dysregulated complement has also been implicated in patients with fibrosis (134). Similar to its involvement in allergy, C3 drives bleomycin-induced mouse models of lung fibrosis (135–137). Additionally, C5 has a dual role in this model: it initially protects against the acute effects of bleomycin by dampening excessive inflammation, but during the chronic phase, it exacerbates fibrosis by promoting collagen deposition (136, 138). These findings suggest that complement not only modulates allergy, but may also play a key role in tissue remodeling.

## C1q and CD46 can protect against allergy

Some proteins of complement have an unambiguous protective effect in allergy. The C1 subunit, C1q, which acts as the initiator of the classical complement pathway, is now appreciated as having regulatory functions independent of the complement cascade. In patients undergoing sublingual allergen therapy, C1q expression is increased in PBMCs of responders compared with those of nonresponders (139). Consistent with this, C1q levels in exhaled breath were significantly lower in patients with poorly controlled asthma as compared with well-controlled asthma (73). Notably, in patients with wasp sting–induced anaphylaxis, levels of serum C1q dropped with concomitant increases in C3 (16). In animals, C1q dampens allergic inflammation and AHR in response to OVA or birch pollen. C1q did not appear to promote protection via Treg expansion but rather through pDC elicitation (140), similar to C5a (121).

CD46, a negative regulator of complement, has also been shown to protect against allergy. CD46 is a regulatory complement membrane protein that binds and acts as a cofactor in the inactivation of opsonins C3b and C4b in humans (141). It is thought that disruption of CD46-mediated Treg induction also contributes to allergic asthma pathogenesis. CD46 costimulation of human CD4^+^ T cells induces the production of IL-10, IFN-γ, and granzyme B, a phenotype consistent with type 1 regulatory T (Tr1) cells (142, 143). This response is impaired in asthma: CD4^+^ T cells isolated from the PBMCs of patients with asthma have impaired production of IL-10 in response to CD3/CD46 stimulation compared with controls (142, 144). The mechanistic basis for failure to induce IL-10 production in the CD4^+^ T cells of individuals with asthma may be attributable to the favored expression of the cytoplasmic (cyt) tail isoform of CD46. CD46-cyt1 favors IL-10, while CD46-cyt2 inhibits IL-10 expression in human CD4^+^ T cells (145). Indeed, CD46-cyt2 expression is higher in individuals with asthma than in control PBMCs, which favor CD46-cyt1 (142). Collectively, these data show that this protective pathway is compromised in susceptible individuals, promoting the development of allergy.

Thus, growing evidence suggests that complement at barrier surfaces may have evolved in vertebrates to interface with acquired immunity to provide crucial context for eliciting durable T cell responses.

## Potential physiological function of the complement-Th2 axis

**Figure 2:**
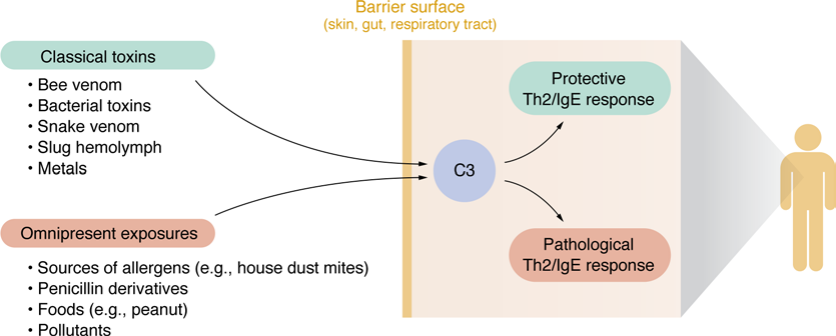
Context-dependent C3 function at barrier sites. C3 functions as an environmental sensor at barrier surfaces, triggering a protective Th2/IgE response to harmful exposures. However, repeated exposure to innocuous proteins (such as allergens and foods), metabolites (like penicillin and its derivatives), or nonimmediate dangers such as air pollution can, in susceptible individuals, lead to pathological Th2/IgE responses.

The evolution of Th2 responses is crucial for eliminating parasitic worms and facilitating tissue repair. However, the response to helminths alone does not fully account for the development of allergies. In addition to its antiparasitic function, the allergic response is believed to have evolved as a defense mechanism against various environmental toxicities (146). It is interesting to consider that complement, a quick-acting system triggered in response to environmental dangers, such as toxic metals, insect and snake venoms, tick bites, bacterial toxins, and snail hemolymph (57, 63, 64, 147–154), would have evolved to act as an adjuvant for the induction of protective, long-lasting Th2 memory and subsequent neutralizing IgE responses against these harmful exposures (155). Based on this, it is tempting to make a parallel to other pathways, such as the mast cell–expressed G protein–coupled receptor MRGPRX2, which also appears to be a unique environmental-sensing system in the skin. MRGPRX2 can be triggered by over 100 different compounds, some of which are toxic, leading to IgE-independent mast cell degranulation (156). Together, these various pathways of mast cell activation may lead to changes in behavior to avoid toxins (157, 158). If we view the complement-Th2 system as an environmental sensor designed to protect us from toxic exposures, we can also anticipate that its repeated activation may increase susceptibility to developing allergies in response to otherwise harmless environmental proteins, such as allergens. Together, this encourages the broader view of allergies to innocuous substances as potential misfires of an immune system that evolved to protect against real dangers (Figure 2).

Beyond Th2-driven allergic responses, complement also influences tissue repair, a critical feature of type 2 immune responses. Although complement can exacerbate pathogenic manifestations of tissue remodeling, such as fibrosis, it also participates in physiological tissue repair. C3 is upregulated in regenerating limbs of axolotl (159) and induces retinal regeneration in chicken (160). Moreover, topical C3 application facilitates wound healing in rats (161), and both C3 and C5 promote bone fracture healing (162). C1q and complement factor D (CFD) also promote collagen expression, angiogenesis, and tissue repair (163–165). However, this pathway is likely tightly regulated, as it can impair wound healing due to overzealous immune cell recruitment (166). While there is evidence that some components of complement can regulate collagen production in vitro, it remains to be discovered whether the prorepair function of complement in vivo occurs by inducing Th2 responses.

## Clinical trials

There has been some exploration of the therapeutic potential for targeting complement in allergic diseases, as summarized in Table 2. In one study, 24 adult individuals with asthma with HDM sensitivity received i.v. administration of C1 inhibitor, an endogenous protein that targets both the lectin and classical pathways (167). This was followed by intrabronchial HDM/LPS challenge in one lung and saline administration in the contralateral lung. The BAL of patients receiving the C1 inhibitor showed reduced levels of C4a and C3a compared with patients receiving the placebo. C1 inhibitor did not abrogate the pulmonary allergic response, as measured by BAL immune cell count (eosinophils, neutrophils, and alveolar macrophages) or bronchoalveolar degranulation of eosinophils and neutrophils. However, C1 inhibitor decreased vascular leak, e.g., reduced BAL IgM. Notably, the concurrent administration of HDM and LPS complicates the interpretation of these findings in the context of allergic disease. LPS can have varied effects on Th2 responses — suppressing them at higher doses while potentiating them at lower doses (168–173). Thus, the potential therapeutic use of C1 inhibitor remains of interest.

In one study, the oral C5aR antagonist NGD 2000–1 demonstrated no improvements in lung function in individuals with asthma (174, 175). However, there has been interest in using the anti-C5 mAb, eculizumab, for treating allergic asthma. Eculizumab is a humanized IgG2/4 k mAb originally approved by the FDA in 2007 for the treatment of paroxysmal nocturnal hemoglobinuria to reduce hemolysis (176). By targeting C5, eculizumab precludes proteolytic activation of C5 by the C5 convertase, thereby preventing the formation of C5a and C5b-9 (177). There is only one published clinical trial to date investigating the potential use of eculizumab in the late asthmatic response (NCT00485576) (178). This phase II trial followed a randomized, double-blind, placebo-controlled crossover study design and enrolled 19 participants with allergen-induced bronchoconstriction. Participants were infused with placebo or 600 mg eculizumab and then exposed to inhaled allergen 24 hours later. A minimum 4-week washout period was considered sufficient for eculizumab levels to drop to noneffective levels, after which participants were administered the opposite treatment. However, a significant period effect was observed, invalidating the study approach. Specifically, an improved late-phase asthmatic response, as measured by area under the curve of forced expiratory volume in 1 second (FEV1) from 3 to 7 hours after challenge, was observed in participants who received placebo first and then eculizumab, supporting drug efficacy. However, participants who received placebo second also had significantly reduced late-asthma response after placebo compared with response at the initial study screening.

Interestingly, prolonged inhibition of C5 activation and reduced IL-13 in sputum was reported for participants receiving anti-C5 as the first treatment and placebo as the second treatment. This unexpected longevity of C5 inhibition could be interpreted to mean that eculizumab was still active; however, the results from this study are inconclusive. Nonetheless, eculizumab’s therapeutic potential for allergic diseases merits further investigation. There are no data on the second-generation iteration of eculizumab, called ravulizumab (179), in the context of allergy.

## Future directions

It is clear that the complement system is more intricate and far-reaching than previously recognized. More than a collection of antimicrobial humoral mediators originating from the liver, it shapes biological processes in unexpected ways. Growing evidence suggests that locally produced complement is critical in health and disease. For instance, C3 activation in synovial fibroblasts induces metabolic changes that prime them for chronic inflammation in arthritis (180). In addition, airway epithelial C3 — distinct from systemic C3 — protects against lung injury in response to bacterial infection (2). These and other studies highlight the need to investigate locally produced complement for their unique contributions to disease and potential therapeutic implications. While we know that there is an increase in local complement during allergy, whether it drives aberrant Th2 responses is unknown. Moreover, allergy often presents as a multitissue disease, where sensitization at one site may predispose development of allergy at a separate site. This may involve a mechanism where local aberrant complement activation at one barrier site may initiate a pathogenic cascade replacing healthy, homeostatic responses to allergen at another. Additionally, whether intracellular complement in the resident cells at barrier sites plays a role in allergic manifestations is unknown. These potential mechanisms of allergenicity represent novel areas of exploration.

Accumulating evidence suggests that local complement activation is a key mechanism linking environmental exposures to the development of allergic diseases. For instance, exposure to pollutants, including microplastics, is a growing source of environmental toxic triggers that drive the accumulation of mucosal complement (106, 181–183). This complement-mediated activation is a potent adjuvant, intensifying the response to otherwise harmless proteins like pollen, dust mites, and pet dander. Studies have shown that particulate matter from air pollution amplifies Th2 responses and IgE production in animals exposed to these allergens, tipping the balance toward an exaggerated immune response. Thus, aberrant complement activation links environmental pollutants directly to the rising prevalence of allergic diseases.

Another intriguing aspect of complement’s role is its potential effect on transmaternal health and early immune development. Breast milk, known to contain complement (184, 185), plays a crucial role in immune modulation during infancy, though its functions remain only partly understood. Evidence suggests that breast milk complement targets Gram-positive bacteria in the infant’s gut, promoting inflammation resolution and barrier integrity (185). Disturbances in the gut microbiome, often linked to complement dysregulation, have been associated with a range of atopic conditions affecting the gut, skin, and lungs. The microbiota in breast milk further influences allergy risk, suggesting that altered complement activity may shape the microbiome in ways that predispose offspring to allergic disease.

In conclusion, the complement system’s expanding roles as a local sensor of environmental exposures — whether venoms, toxins, metals, allergens, or microbes — directly impact barrier site health, dysregulation of which contributes to the development of allergy.

## Figures and Tables

**Table 2:**
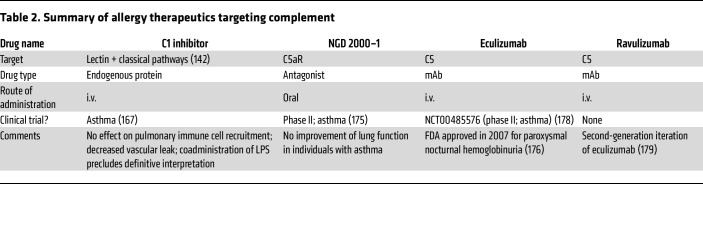
Summary of allergy therapeutics targeting complement

**Table 1:**
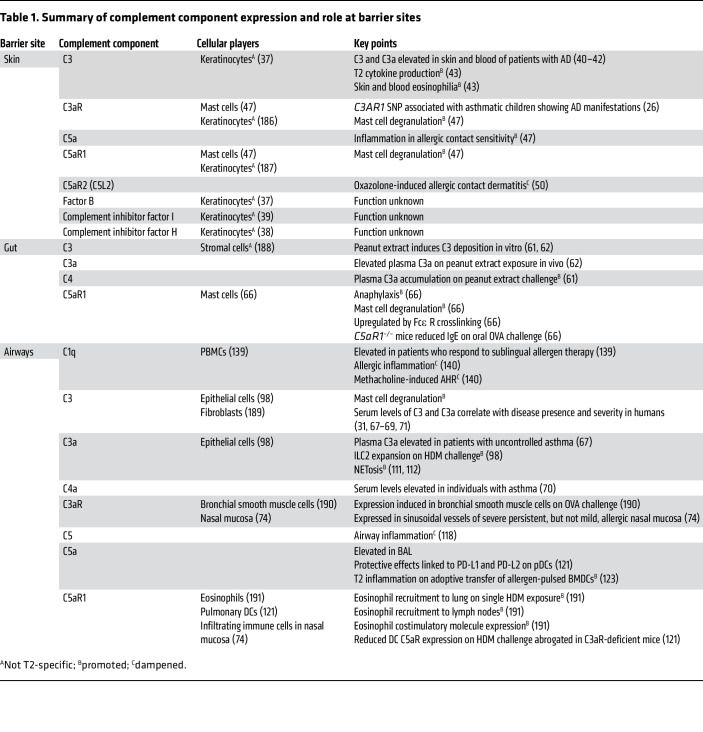
Summary of complement component expression and role at barrier sites

## Version 1 05/01/2025

Electronic publication

## References

[1] Mastellos DC (2024). A guide to complement biology, pathology and therapeutic opportunity. Nat Rev Immunol.

[2] Sahu SK (2023). Lung epithelial cell-derived C3 protects against pneumonia-induced lung injury. Sci Immunol.

[3] Morgan BP, Gasque P (2003). Extrahepatic complement biosynthesis: where, when and why?. Clin Exp Immunol.

[4] Liszewski MK (2013). Intracellular complement activation sustains T cell homeostasis and mediates effector differentiation. Immunity.

[5] West EE, Kemper C (2023). Complosome — the intracellular complement system. Nat Rev Nephrol.

[6] King BC, Blom AM (2023). Intracellular complement: Evidence, definitions, controversies, and solutions. Immunol Rev.

[7] Freeley S (2018). Asparaginyl endopeptidase (legumain) supports human Th1 induction via cathepsin L-mediated intracellular C3 activation. Front Immunol.

[8] Sorbara MT (2018). Complement C3 drives autophagy-dependent restriction of cyto-invasive bacteria. Cell Host Microbe.

[9] King BC (2019). Complement component C3 is highly expressed in human pancreatic islets and prevents beta cell death via ATG16L1 interaction and autophagy regulation. Cell Metab.

[10] Kolev M (2020). Diapedesis-induced integrin signaling via LFA-1 facilitates tissue immunity by inducing intrinsic complement C3 expression in immune cells. Immunity.

[11] https://www.cdc.gov/asthma/most_recent_national_asthma_data.htm.

[12] Ng A, Boersma P (2023). Diagnosed allergic conditions in adults: United States, 2021. NCHS Data Brief.

[13] Zablotsky B (2023). Diagnosed allergic conditions in children aged 0-17 years: United States, 2021. NCHS Data Brief.

[14] Kharaba Z (2022). An assessment of quality of life in patients with asthma through physical, emotional, social, and occupational aspects. A cross-sectional study. Front Public Health.

[15] Andrew N, Booth T (1991). The social impact of asthma. Fam Pract.

[16] Kopp EB (2023). Modes of type 2 immune response initiation. Immunity.

[17] Hill DA, Spergel JM (2018). The atopic march. Ann Allergy Asthma Immunol.

[18] Thyssen JP (2023). Comorbidities of atopic dermatitis-what does the evidence say?. J Allergy Clin Immunol.

[19] Yoo Y, Perzanowski MS (2014). Allergic sensitization and the environment: Latest update. Curr Allergy Asthma Rep.

[20] Kaufmann SH (2008). Immunology’s foundation: the 100-year anniversary of the Nobel Prize to Paul Ehrlich and Elie Metchnikoff. Nat Immunol.

[21] Reis ES (2019). New insights into the immune functions of complement. Nat Rev Immunol.

[22] Klos A (2009). The role of the anaphylatoxins in health and disease. Mol Immunol.

[23] Finkelman FD (2016). Human IgE-independent systemic anaphylaxis. J Allergy Clin Immunol.

[24] Undarmaa S (2010). Replication of genetic association studies in asthma and related phenotypes. J Hum Genet.

[25] Inoue H (2008). Association study of the C3 gene with adult and childhood asthma. J Hum Genet.

[26] Hasegawa K (2004). Variations in the C3, C3a receptor, and C5 genes affect susceptibility to bronchial asthma. Hum Genet.

[27] Barnes KC (2006). Variants in the gene encoding C3 are associated with asthma and related phenotypes among African Caribbean families. Genes Immun.

[28] Berube JC (2016). Identification of susceptibility genes of adult asthma in French Canadian women. Can Respir J.

[29] Messelodi D (2022). *C5* and *SRGAP3* polymorphisms are linked to paediatric allergic asthma in the Italian population. Genes (Basel).

[30] Purwar R (2009). Polymorphisms within the C3 gene are associated with specific IgE levels to common allergens and super-antigens among atopic dermatitis patients. Exp Dermatol.

[31] Vedel-Krogh S (2021). Complement C3 and allergic asthma: a cohort study of the general population. Eur Respir J.

[32] Afshari M (2024). Unraveling the skin; a comprehensive review of atopic dermatitis, current understanding, and approaches. Front Immunol.

[33] Arents B (2022). Systemic immunomodulatory treatments for atopic dermatitis: update of a living systematic review and network meta-analysis. JAMA Dermatol.

[34] Tham EH (2020). Epicutaneous sensitization to food allergens in atopic dermatitis: What do we know?. Pediatr Allergy Immunol.

[35] Beck LA, Leung DYM (2000). Allergen sensitization through the skin induces systemic allergic responses. J Allergy Clin Immunol.

[36] Klicznik MM (2018). Taking the lead - how keratinocytes orchestrate skin T cell immunity. Immunol Lett.

[37] Pasch MC (2000). Synthesis of complement components C3 and factor B in human keratinocytes is differentially regulated by cytokines. J Invest Dermatol.

[38] Timar KK (2006). Human keratinocytes produce the complement inhibitor factor H: synthesis is regulated by interferon-gamma. Mol Immunol.

[39] Timar KK (2007). Human keratinocytes produce the complement inhibitor factor I: synthesis is regulated by interferon-gamma. Mol Immunol.

[40] Kapp A (1985). Involvement of complement in psoriasis and atopic dermatitis--measurement of C3a and C5a, C3, C4 and C1 inactivator. Arch Dermatol Res.

[41] Ring J (1978). Complement and immunoglobulin deposits in the skin of patients with atopic dermatitis. Br J Dermatol.

[42] Kaufman HS (1968). Serum complement (_β1_C) in young children with atopic dermatitis. J Allergy.

[43] Yalcindag A (2006). The complement component C3 plays a critical role in both Th1 and Th2 responses to antigen. J Allergy Clin Immunol.

[44] Giang J (2018). Complement activation in inflammatory skin diseases. Front Immunol.

[45] Kasperska-Zajac A (2013). Increased serum complement C3 and C4 concentrations and their relation to severity of chronic spontaneous urticaria and CRP concentration. J Inflamm (Lond).

[46] Magen E (2019). Factors related to omalizumab resistance in chronic spontaneous urticaria. Allergy Asthma Proc.

[47] Schafer B (2013). Mast cell anaphylatoxin receptor expression can enhance IgE-dependent skin inflammation in mice. J Allergy Clin Immunol.

[48] Niebuhr M (2012). Participation of complement 3a receptor (C3aR) in the sensitization phase of Th2 mediated allergic contact dermatitis. Exp Dermatol.

[49] Purwar R (2011). A protective role of complement component 3 in T cell-mediated skin inflammation. Exp Dermatol.

[50] Wang R (2013). Disruption of the complement anaphylatoxin receptor C5L2 exacerbates inflammation in allergic contact dermatitis. J Immunol.

[51] Bamberg CE (2010). The C5a receptor (C5aR) C5L2 is a modulator of C5aR-mediated signal transduction. J Biol Chem.

[52] Midander K (2023). Cobalt nanoparticles cause allergic contact dermatitis in humans. Br J Dermatol.

[53] Rostenberg A (1951). , Perkins AJ. Nickel and cobalt dermatitis. J Allergy.

[54] Fonacier L (2024). Recognizing and managing allergic contact dermatitis: focus on major allergens. J Allergy Clin Immunol Pract.

[55] Kolberg L (2020). Nickel allergy is associated with wheezing and asthma in a cohort of young German adults: results from the SOLAR study. ERJ Open Res.

[56] Brera S, Nicolini A (2005). Respiratory manifestations due to nickel. Acta Otorhinolaryngol Ital.

[57] Acevedo F, Vesterberg O (2003). Nickel and cobalt activate complement factor C3 faster than magnesium. Toxicology.

[58] Gupta RS (2019). Prevalence and severity of food allergies among US adults. JAMA Netw Open.

[59] Warren CM (2023). The epidemiology of multifood allergy in the United States: A population-based study. Ann Allergy Asthma Immunol.

[60] Brown SG (2013). Anaphylaxis: clinical patterns, mediator release, and severity. J Allergy Clin Immunol.

[61] Kodama T (2013). Role of complement in a murine model of peanut-induced anaphylaxis. Immunobiology.

[62] Khodoun M (2009). Peanuts can contribute to anaphylactic shock by activating complement. J Allergy Clin Immunol.

[63] van der Linden PW (1990). Preliminary report: complement activation in wasp-sting anaphylaxis. Lancet.

[64] De Carolis C (1982). Complement activation by Hymenoptera venom allergenic extracts. J Allergy Clin Immunol.

[65] Szymański Ł (2022). Time-dependent effect of desensitization with wasp venom on selected parameters of the immune system. Sci Rep.

[66] Kordowski A (2019). C5a receptor 1^-/-^ mice are protected from the development of IgE-mediated experimental food allergy. Allergy.

[67] Nakano Y (2003). Elevated complement C3a in plasma from patients with severe acute asthma. J Allergy Clin Immunol.

[68] Vedel-Krogh S (2021). Complement C3 and allergic asthma: a cohort study of the general population. Eur Respir J.

[69] Liu W (2017). Mechanism of T_H_2/T_H_17-predominant and neutrophilic T_H_2/T_H_17-low subtypes of asthma. J Allergy Clin Immunol.

[70] Lee SH (2006). Complement C3a and C4a increased in plasma of patients with aspirin-induced asthma. Am J Respir Crit Care Med.

[71] Fattah MA (2010). Complement components (C3, C4)# as inflammatory markers in asthma. Indian J Pediatr.

[72] Marc MM (2004). Complement factors C3a, C4a, and C5a in chronic obstructive pulmonary disease and asthma. Am J Respir Cell Mol Biol.

[73] Kokelj S (2023). Activation of the complement and coagulation systems in the small airways in asthma. Respiration.

[74] Jun SW (2008). Overexpression of the anaphylatoxin receptors, complement anaphylatoxin 3a receptor and complement anaphylatoxin 5a receptor, in the nasal mucosa of patients with mild and severe persistent allergic rhinitis. J Allergy Clin Immunol.

[75] Werner U (2020). Linking complement C3 and B cells in nasal polyposis. J Immunol Res.

[76] Fregonese L (2005). Expression of the anaphylatoxin receptors C3aR and C5aR is increased in fatal asthma. J Allergy Clin Immunol.

[77] Krug N (2001). Complement factors C3a and C5a are increased in bronchoalveolar lavage fluid after segmental allergen provocation in subjects with asthma. Am J Respir Crit Care Med.

[78] Humbles AA (2000). A role for the C3a anaphylatoxin receptor in the effector phase of asthma. Nature.

[79] Weiszhar Z (2013). Elevated complement factor H levels in asthmatic sputa. J Clin Immunol.

[80] Nishioka T (2008). Alpha-1-antitrypsin and complement component C7 are involved in asthma exacerbation. Proteomics Clin Appl.

[81] Wu J (2005). Differential proteomic analysis of bronchoalveolar lavage fluid in asthmatics following segmental antigen challenge. Mol Cell Proteomics.

[82] Taube C (2006). Factor B of the alternative complement pathway regulates development of airway hyperresponsiveness and inflammation. Proc Natl Acad Sci U S A.

[83] Rocha ESM (1951). Histamine release by anaphylatoxin. Nature.

[84] Lepow IH (1970). Gross and ultrastructural observations on lesions produced by intradermal injection of human C3a in man. Am J Pathol.

[85] Stimler NP (1983). C3a-induced contraction of guinea pig lung parenchyma: role of cyclooxygenase metabolites. Immunopharmacology.

[86] Hawlisch H (2004). The anaphylatoxins bridge innate and adaptive immune responses in allergic asthma. Mol Immunol.

[87] Zaidi AK (2006). Response to C3a, mast cells, and asthma. FASEB J.

[88] Bradding P (2005). C3a, mast cells, and asthma. FASEB J.

[89] Thangam EB (2005). Airway smooth muscle cells enhance C3a-induced mast cell degranulation following cell-cell contact. FASEB J.

[90] Tainsh KR (1992). Mast cell heterogeneity in man: unique functional properties of skin mast cells in response to a range of polycationic stimuli. Immunopharmacology.

[91] Schulman ES (1988). Differential effects of the complement peptides, C5a and C5a des Arg on human basophil and lung mast cell histamine release. J Clin Invest.

[92] el-Lati SG (1994). Complement peptides C3a- and C5a-induced mediator release from dissociated human skin mast cells. J Invest Dermatol.

[93] Lawrence ID (1987). Purification and characterization of human skin mast cells. Evidence for human mast cell heterogeneity. J Immunol.

[94] Fureder W (1995). Differential expression of complement receptors on human basophils and mast cells. Evidence for mast cell heterogeneity and CD88/C5aR expression on skin mast cells. J Immunol.

[95] Irani AA (1986). Two types of human mast cells that have distinct neutral protease compositions. Proc Natl Acad Sci U S A.

[96] Brightling CE (2002). Mast-cell infiltration of airway smooth muscle in asthma. N Engl J Med.

[97] Linton S (2023). Evidence-based use of antihistamines for treatment of allergic conditions. Ann Allergy Asthma Immunol.

[98] Gour N (2018). C3a is required for ILC2 function in allergic airway inflammation. Mucosal Immunol.

[99] Roy RM (2013). Complement component 3C3 and C3a receptor are required in chitin-dependent allergic sensitization to Aspergillus fumigatus but dispensable in chitin-induced innate allergic inflammation. mBio.

[100] Drouin SM (2002). Absence of the complement anaphylatoxin C3a receptor suppresses Th2 effector functions in a murine model of pulmonary allergy. J Immunol.

[101] Lajoie S (2010). Complement-mediated regulation of the IL-17A axis is a central genetic determinant of the severity of experimental allergic asthma. Nat Immunol.

[102] Mulligan JK (2018). C3a receptor antagonism as a novel therapeutic target for chronic rhinosinusitis. Mucosal Immunol.

[103] Bautsch W (2000). Cutting edge: guinea pigs with a natural C3a-receptor defect exhibit decreased bronchoconstriction in allergic airway disease: evidence for an involvement of the C3a anaphylatoxin in the pathogenesis of asthma. J Immunol.

[104] Koren HS (1989). Ozone-induced inflammation in the lower airways of human subjects. Am Rev Respir Dis.

[105] Polack FP (2002). A role for immune complexes in enhanced respiratory syncytial virus disease. J Exp Med.

[106] Walters DM (2002). Complement factor 3 mediates particulate matter-induced airway hyperresponsiveness. Am J Respir Cell Mol Biol.

[107] Zheng Z (2021). Lung mesenchymal stromal cells influenced by Th2 cytokines mobilize neutrophils and facilitate metastasis by producing complement C3. Nat Commun.

[108] Sanchez-Guzman D (2021). Long-term evolution of the epithelial cell secretome in preclinical 3D models of the human bronchial epithelium. Sci Rep.

[109] Li K (2008). Cyclic AMP plays a critical role in C3a-receptor-mediated regulation of dendritic cells in antigen uptake and T-cell stimulation. Blood.

[110] Engelke C (2014). Distinct roles of the anaphylatoxins C3a and C5a in dendritic cell-mediated allergic asthma. J Immunol.

[111] Wu X (2023). Knockout of the C3a receptor protects against renal ischemia reperfusion injury by reduction of NETs formation. Cell Mol Life Sci.

[112] Wu X (2022). Reduced neutrophil extracellular trap formation during ischemia reperfusion injury in C3 KO mice: C3 requirement for NETs release. Front Immunol.

[113] Gal Z (2020). Plasma neutrophil extracellular trap level is modified by disease severity and inhaled corticosteroids in chronic inflammatory lung diseases. Sci Rep.

[114] Curren B (2023). IL-33-induced neutrophilic inflammation and NETosis underlie rhinovirus-triggered exacerbations of asthma. Mucosal Immunol.

[115] Chacon P (2024). Human neutrophils couple nitric oxide production and extracellular trap formation in allergic asthma. Am J Respir Crit Care Med.

[116] Dillard P (2007). Complement C3a regulates Muc5ac expression by airway Clara cells independently of Th2 responses. Am J Respir Crit Care Med.

[117] Mulligan JK (2022). Role of C3a as a novel regulator of 25(OH)D_3_ to 1,25(OH)_2_D_3_ metabolism in upper airway epithelial cells. J Immunol.

[118] Karp CL (2000). Identification of complement factor 5 as a susceptibility locus for experimental allergic asthma. Nat Immunol.

[119] Kohl J (2006). A regulatory role for the C5a anaphylatoxin in type 2 immunity in asthma. J Clin Invest.

[120] Baelder R (2005). Pharmacological targeting of anaphylatoxin receptors during the effector phase of allergic asthma suppresses airway hyperresponsiveness and airway inflammation. J Immunol.

[121] Zhang X (2009). A protective role for C5a in the development of allergic asthma associated with altered levels of B7-H1 and B7-DC on plasmacytoid dendritic cells. J Immunol.

[122] Kool M (2009). An anti-inflammatory role for plasmacytoid dendritic cells in allergic airway inflammation. J Immunol.

[123] Schmudde I (2013). C5a receptor signalling in dendritic cells controls the development of maladaptive Th2 and Th17 immunity in experimental allergic asthma. Mucosal Immunol.

[124] Wiese AV (2017). The C5a/C5aR1 axis controls the development of experimental allergic asthma independent of LysM-expressing pulmonary immune cells. PLoS One.

[125] Gutzmer R (2006). Human plasmacytoid dendritic cells express receptors for anaphylatoxins C3a and C5a and are chemoattracted to C3a and C5a. J Invest Dermatol.

[126] McCubbrey AL (2017). Promoter specificity and efficacy in conditional and inducible transgenic targeting of lung macrophages. Front Immunol.

[127] Wiese AV (2023). C5aR1 activation in mice controls inflammatory eosinophil recruitment and functions in allergic asthma. Allergy.

[128] Strainic MG (2013). Absence of signaling into CD4^+^ cells via C3aR and C5aR enables autoinductive TGF-β1 signaling and induction of Foxp3+ regulatory T cells. Nat Immunol.

[129] Kwan WH (2013). Signaling through C5a receptor and C3a receptor diminishes function of murine natural regulatory T cells. J Exp Med.

[130] Lim H (2012). Negative regulation of pulmonary Th17 responses by C3a anaphylatoxin during allergic inflammation in mice. PLoS One.

[131] Mizutani N (2012). Complement C3a-induced IL-17 plays a critical role in an IgE-mediated late-phase asthmatic response and airway hyperresponsiveness via neutrophilic inflammation in mice. J Immunol.

[132] Asgari E (2013). C3a modulates IL-1β secretion in human monocytes by regulating ATP efflux and subsequent NLRP3 inflammasome activation. Blood.

[133] (2010). , et al. C5a receptor-deficient dendritic cells promote induction of Treg and Th17 cells. Eur J Immunol.

[134] Dreisin RB (1978). Circulating immune complexes in the idiopathic interstitial pneumonias. N Engl J Med.

[135] Fisher AJ (2017). Potential mechanisms underlying TGF-β-mediated complement activation in lung fibrosis. Cell Mol Med Open Access.

[136] Gu H (2016). Contribution of the anaphylatoxin receptors, C3aR and C5aR, to the pathogenesis of pulmonary fibrosis. FASEB J.

[137] Gu H (2014). Crosstalk between TGF-β1 and complement activation augments epithelial injury in pulmonary fibrosis. FASEB J.

[138] Addis-Lieser E (2005). Opposing regulatory roles of complement factor 5 in the development of bleomycin-induced pulmonary fibrosis. J Immunol.

[139] Zimmer A (2012). A regulatory dendritic cell signature correlates with the clinical efficacy of allergen-specific sublingual immunotherapy. J Allergy Clin Immunol.

[140] Mascarell L (2017). The regulatory dendritic cell marker C1q is a potent inhibitor of allergic inflammation. Mucosal Immunol.

[141] Liszewski MK (2005). Emerging roles and new functions of CD46. Springer Semin Immunopathol.

[142] Tsai YG (2012). Functional defects of CD46-induced regulatory T cells to suppress airway inflammation in mite allergic asthma. Lab Invest.

[143] Sánchez A (2004). CD46-mediated costimulation induces a Th1-biased response and enhances early TCR/CD3 signaling in human CD4+ T lymphocytes. Eur J Immunol.

[144] Xu Y-Q (2010). A defect of CD4^+^CD25^+^ regulatory T cells in inducing interleukin-10 production from CD^+^ T cells under CD46 costimulation in asthma patients. J Asthma.

[145] Marie JC (2002). Linking innate and acquired immunity: divergent role of CD46 cytoplasmic domains in T cell induced inflammation. Nat Immunol.

[146] Profet M (1991). The function of allergy: immunological defense against toxins. Q Rev Biol.

[147] Arvidsson I (2015). Shiga toxin-induced complement-mediated hemolysis and release of complement-coated red blood cell-derived microvesicles in hemolytic uremic syndrome. J Immunol.

[148] Leonel TB (2022). *Bothrops jararaca* snake venom inflammation induced in human whole blood: role of the complement system. Front Immunol.

[149] Nicolson IC (1974). Boomslang bite with haemorrhage and activation of complement by the alternate pathway. Clin Exp Immunol.

[150] Birdsey V (1971). Interaction of toxic venoms with the complement system. Immunology.

[151] Shoemaker RC (2008). Complement split products C3a and C4a are early markers of acute Lyme disease in tick bite patients in the United States. Int Arch Allergy Immunol.

[152] Rantanen T (1982). Persistent pruritic papules from deer ked bites. Acta Derm Venereol.

[153] Koch C, Nielsen HE (1984). Activation of vertebrate complement by Helix pomatia haemolymph. Dev Comp Immunol.

[154] Kurpiewski G (1981). Alternate complement pathway activation by recluse spider venom. Int J Tissue React.

[155] Palm NW (2013). Bee venom phospholipase A2 induces a primary type 2 response that is dependent on the receptor ST2 and confers protective immunity. Immunity.

[156] Gour N, Dong X (2024). The MRGPR family of receptors in immunity. Immunity.

[157] Plum T (2023). Mast cells link immune sensing to antigen-avoidance behaviour. Nature.

[158] Florsheim EB (2023). Immune sensing of food allergens promotes avoidance behaviour. Nature.

[159] Del Rio-Tsonis K (1998). Expression of the third component of complement, C3, in regenerating limb blastema cells of urodeles. J Immunol.

[160] Haynes T (2013). Complement anaphylatoxin C3a is a potent inducer of embryonic chick retina regeneration. Nat Commun.

[161] Sinno H (2013). Topical application of complement C3 in collagen formulation increases early wound healing. J Dermatolog Treat.

[162] Ehrnthaller C (2013). Complement C3 and C5 deficiency affects fracture healing. PLoS One.

[163] Hayuningtyas RA (2021). The collagen structure of C1q induces wound healing by engaging discoidin domain receptor 2. Mol Med.

[164] Chen J (2023). Complement factor D regulates collagen type I expression and fibroblast migration to enhance human tendon repair and healing outcomes. Front Immunol.

[165] Bossi F (2014). C1q as a unique player in angiogenesis with therapeutic implication in wound healing. Proc Natl Acad Sci U S A.

[166] Rafail S (2015). Complement deficiency promotes cutaneous wound healing in mice. J Immunol.

[167] Yang J (2020). Effect of C1-inhibitor in adults with mild asthma: A randomized controlled trial. Allergy.

[168] Gerhold K (2002). Endotoxins prevent murine IgE production, T(H)2 immune responses, and development of airway eosinophilia but not airway hyperreactivity. J Allergy Clin Immunol.

[169] Zakeri A, Russo M (2018). Dual role of toll-like receptors in human and experimental asthma models. Front Immunol.

[170] Hammad H (2009). House dust mite allergen induces asthma via Toll-like receptor 4 triggering of airway structural cells. Nat Med.

[171] Eisenbarth SC (2002). Lipopolysaccharide-enhanced, toll-like receptor 4-dependent T helper cell type 2 responses to inhaled antigen. J Exp Med.

[172] Daan de Boer J (2013). Lipopolysaccharide inhibits Th2 lung inflammation induced by house dust mite allergens in mice. Am J Respir Cell Mol Biol.

[173] Schuijs MJ (2015). Farm dust and endotoxin protect against allergy through A20 induction in lung epithelial cells. Science.

[174] Monk PN (2007). Function, structure and therapeutic potential of complement C5a receptors. Br J Pharmacol.

[175] Zhang X, Köhl J (2010). A complex role for complement in allergic asthma. Expert Rev Clin Immunol.

[176] Rother RP (2007). Discovery and development of the complement inhibitor eculizumab for the treatment of paroxysmal nocturnal hemoglobinuria. Nat Biotechnol.

[177] Smith SG (2012). Eculizumab for treatment of asthma. Expert Opin Biol Ther.

[178] Gavreau G (2009). The effect of C5 inhibition by eculizumab on allergen-induced asthmatic responses in patients. Allergy.

[179] Garred P (2021). Therapeutic targeting of the complement system: from rare diseases to pandemics. Pharmacol Rev.

[180] Friščić J (2021). The complement system drives local inflammatory tissue priming by metabolic reprogramming of synovial fibroblasts. Immunity.

[181] Davis KS (2010). Murine complement deficiency ameliorates acute cigarette smoke-induced nasal damage. Otolaryngol Head Neck Surg.

[182] Yuan Y (2023). Acute polyethylene microplastic (PE-MPs) exposure activates the intestinal mucosal immune network pathway in adult zebrafish (Danio rerio). Chemosphere.

[183] Liang Y (2024). Polystyrene microplastics induce kidney injury via gut barrier dysfunction and C5a/C5aR pathway activation. Environ Pollut.

[184] Ogundele MO (2000). Activation and deposition of human breast-milk complement C3 opsonins on serum sensitive Escherichia coli 0111. J Reprod Immunol.

[185] Xu D (2024). Complement in breast milk modifies offspring gut microbiota to promote infant health. Cell.

[186] Mommert S (2021). C3a and its receptor C3aR are detectable in normal human epidermal keratinocytes and are differentially regulated via TLR3 and LL37. J Innate Immun.

[187] Fayyazi A (1999). C5a receptor and interleukin-6 are expressed in tissue macrophages and stimulated keratinocytes but not in pulmonary and intestinal epithelial cells. Am J Pathol.

[188] Wu M (2024). Gut complement induced by the microbiota combats pathogens and spares commensals. Cell.

[189] Atakkatan A (2023). Identification and function of a C3^hi^ IL-33^+^ fibroblast population in the lungs. J Immunol.

[190] Drouin SM (2001). Expression of the complement anaphylatoxin C3a and C5a receptors on bronchial epithelial and smooth muscle cells in models of sepsis and asthma. J Immunol.

[191] Wiese AV (2023). C5aR1 activation in mice controls inflammatory eosinophil recruitment and functions in allergic asthma. Allergy.

